# Exploratory mass spectrometry of cerebrospinal fluid from persons with autopsy-confirmed LATE-NC

**DOI:** 10.21203/rs.3.rs-3252238/v1

**Published:** 2023-08-22

**Authors:** Jozsef Gal, Calvin Vary, Carlos A. Gartner, Gregory A. Jicha, Erin L. Abner, Yulica S. Ortega, Ibrahim Choucair, Donna M. Wilcock, Ruth Nelson, Peter Nelson

**Affiliations:** University of Kentucky; University of Kentucky; University of Kentucky; University of Kentucky; University of Kentucky; University of Kentucky; University of Kentucky; Indiana University; Emory University; University of Kentucky

**Keywords:** RET4, RBP4, CNDP1, aging, adipokine, BRI2

## Abstract

**Background:**

Common neuropathologies associated with dementia include Alzheimer’s disease neuropathologic change (ADNC) and limbic-predominant age-related TDP-43 encephalopathy neuropathologic change (LATE-NC). Biofluid proteomics provides a window into the pathobiology of dementia and the information from biofluid tests may help guide clinical management.

**Methods:**

Participants were recruited from a longitudinal cohort of older adults at the University of Kentucky AD Research Center. A convenience sample of clinically obtained lumbar puncture cerebrospinal fluid (CSF) samples was analyzed from 29 older adults that had autopsy confirmation of the presence or absence of LATE-NC. Nine of the participants had autopsy-confirmed LATE-NC. Antemortem CSF specimens were analyzed in two separate processes: From one group, aliquots were depleted of highly abundant proteins using affinity spin columns. Tryptic digests of sample proteins were subjected to liquid chromatographic separation and mass spectrometry using an Eksigent Ekspert nanoLC 400 system in line with a Sciex 6600+ mass spectrometer. Protein identification was performed using Protein Pilot (Sciex, ver. 5) software, and relative quantification was performed using the SWATH processing microApp in PeakView and MarkerView software (Sciex), respectively. Following data analyses, additional studies were performed using western blots.

**Results:**

A total of 830 proteins were identified in the samples depleted of abundant proteins, and 730 proteins were identified in the non-depleted samples. Whereas some dementia-related proteins were detected (Aβ peptide and α-synuclein protein), others were not (TDP-43, TMEM106B, and tau proteins). When the Bonferroni correction was applied to correct for multiple comparisons, only 4 proteins showed differential expression (LATE-NC vs non-LATE-NC) in the nondepleted samples (RBP4, MIF, IGHG3 and ITM2B), whereas none showed statistically different changes in the depleted samples. Post-hoc western blots confirmed that RBP4 expression was higher in the LATE-NC cases at the group level, but there was overlap between the levels of RBP4 in LATE-NC and non-LATE-NC cases.

**Conclusions:**

An exploratory assessment of CSF proteomes of autopsy-confirmed LATE-NC and non-LATE-NC cases from a community-based cohort failed to demonstrate a clear-cut proteomic fingerprint that distinguished the two groups. There was intriguing increase in RBP4 protein levels in CSF from LATE-NC cases. This may provide clues about pathogenetic mechanisms in LATE-NC.

## Introduction

Dementia is a heterogeneous clinical syndrome with a large impact on public health. The key clinical features of dementia are deficits of memory and/or cognition, such that functional capacity is impaired. However, antemortem biomarker studies have demonstrated that many pathological changes are detectable well before the onset of the severe symptoms and eventual death/autopsy.

Biofluid proteomics provides a potentially vital window into the pathobiology of dementia. The biofluids most commonly assessed in dementia-related research are blood and cerebrospinal fluid (CSF). One goal of biomarkers is to predict the neuropathological status of a person’s brain during life. In the near future, the information from biofluid tests is anticipated to also help guide clinical management and prognostic education and support for patients and families.

The neuropathologic features associated with dementia include Alzheimer’s disease neuropathologic change (ADNC), limbic-predominant age-related TDP-43 encephalopathy neuropathologic change (LATE-NC), Lewy body pathologies (LBP) and cerebrovascular diseases among others (1–4). Together, non-ADNC pathologic features have been demonstrated to account for > 50% of AD-type dementia risk in a large community-based cohort (5, 6). At present, there are FDA-approved tests for indicating ADNC but *in vivo* detection of other pathologies represents a great unmet need that is only beginning to be addressed.

LATE-NC is a common and under-appreciated driver of cognitive impairment in aging. At present, LATE can only be definitively diagnosed at autopsy. TDP-43 pathology was discovered as a disease-related diagnosable feature in the context of frontotemporal lobar degeneration (FTLD-TDP) and amyotrophic lateral sclerosis (7). However, TDP-43 pathology is now known to be present in multiple additional neurologic disease states (8). LATE-NC describes a stereotypic pattern of TDP-43 pathology that sequentially affects the amygdala region (Stage 1), then the hippocampal region (Stage 2), and then expands more broadly to ultimately also involve the middle frontal gyrus (Stage 3) in some cases (2, 9, 10). Given its relatively limited distribution in the brain, and hence potentially low levels of LATE-NC associated analytes, the detection of biofluid analytes that may be diagnostic for LATE-NC has remained problematic.

Immunoassays for TDP-43 protein in cerebrospinal fluid and blood have some promise as a marker of TDP-43 pathologies. In a recent study of human plasma samples, TDP-43 protein was found to be in glial-derived exosomes, which may provide a much-needed blood-based biomarker for LATE-NC (11). However, we note that other disruptions occur in LATE-NC and much remains to be learned about LATE-NC. Although TDP-43 pathology is what is used to diagnose LATE-NC, it is possible that other proteins are disrupted in the course of the disease. More specifically, along with TDP-43 proteinopathy, other proteinaceous and vasculopathic changes have been associated with LATE pathologic phenotypes (12–16). It may be desirable to broaden the scope of study for LATE-NC molecular pathology, because if there are other dysregulated proteins in the disease, that could provide both diagnostic information as well as aid in the identification of alternative potential therapeutic targets.

In the current study, we performed an exploratory proteomics (mass spectrometry) analysis of CSF obtained via lumbar puncture during life, in individuals who eventually came to autopsy. Our study was designed to detect whether persons with substantial LATE-NC (Stage > 1) can be identified during life based on the protein/peptide composition of lumbar puncture-derived CSF using LC-MS proteomic analysis techniques.

## Methods

### Subjects and CSF draws

All participants provided informed consent under a protocol approved by the Institutional Review Board of the University of Kentucky (UK). Participants were recruited from an existing longitudinal cohort of older adults spanning the cognitive continuum at UK’s Sanders-Brown Center on Aging (SBCoA). Information about recruitment, exclusion/inclusion criteria, and other aspects of the cohort are conveyed in prior studies (17–19). Briefly: Exclusionary criteria for enrollment into the SBCoA cohort include a past history of major head injury; major stroke, major psychiatric illness, or current substance abuse; and or encephalitis; meningitis; or epilepsy. Cases with both available CSF drawn during life and subsequent autopsy evaluation that included TDP-43 workup were included. Antemortem lumbar puncture-derived CSF samples from 29 older adults were analyzed in this exploratory analysis.

CSF was drawn the morning after fasting since midnight according to current ADRC best practices/National Institute on Aging guidelines (https://www.alz.washington.edu/BiospecimenTaskForce.html).) CSF was collected using a 20-gauge needle, 15 mL sterile polypropylene collection tubes, and was stored in single-use 0.5 mL aliquots in polypropylene storage tubes at − 80°C.

### Depletion of highly abundant CSF proteins (for one-half of the study).

From each CSF sample tube, 200 µl aliquots were depleted of highly abundant proteins. The abundant proteins (albumin, IgA, IgD, IgE, IgG, kappa and lambda light chains, IgM, α1-acid glycoprotein, α1-antitrypsin, α2-macroglobulin, apolipoprotein A1, fibrinogen, haptoglobin, and transferrin) were depleted with the High-Select Top14 Abundant Protein Depletion Mini Spin Columns (Thermo Fisher Scientific, Cat. # A36370). Preliminary experiments showed that each cartridge can effectively deplete 200 µl of the CSF samples. The depletion resins in the cartridges were suspended into the storage buffer, removed from the cartridge, and transferred into Low Protein Binding Microcentrifuge Tubes (Thermo Fisher Scientific, 90410). The tubes were centrifuged at 1,000 × g for 1 minute at room temperature, and the supernatants were discarded. Each resin was washed with 1 ml 1× PBS (137 mM NaCl, 2.7 mM KCl, 10 mM Na_2_HPO_4_, 1.8 mM KH_2_PO_4_, pH 7.4), centrifuged at 1,000 × g for 1 minute at room temperature, and the supernatants were discarded. To each tube, 200 µl CSF sample was added, and the tubes were incubated at room temperature for 20 minutes with end-over-end rotation. The suspensions were transferred back into the kit-provided filter cartridges, and the depleted CSF samples were collected by centrifugation at 1,000 × g for 2 minutes at room temperature. The depleted CSF samples, and also the non-depleted portions of each CSF sample tube, were frozen on dry ice.

### Sample Preparation for LC-MS/MS

Following thawing from dry ice, protein concentration was measured for each sample using the Pierce Bicinchoninic acid (BCA) assay (Thermo Fisher Scientific, Waltham, MA). An estimated 50 µg protein from each sample was diluted with 8.0 M urea (proteomics grade, MP Biomedicals, Santa Ana, CA) containing 100 mM ammonium bicarbonate (ABC, proteomics grade, MP Biomedicals) to bring the urea concentration below 6.0 M. Tris (2-carboxyethyl) phosphine (TCEP, proteomics grade, Strem Chemicals, Newburyport, MA) was added from a 300 mM stock solution prepared in 1.0 M Tris base (Ultrapure grade, Alfa Aesar, Haverhill, MA) to a final concentration of 10 mM and the samples reduced at 55°C for 20 minutes. After cooling to room temperature, iodoacetamide (G-Biosciences, St. Louis, MO) was added from a 0.5 M stock solution prepared in water to a final concentration of 25 mM. Alkylation was allowed to proceed in the dark for 20 minutes at room temperature before precipitating protein from each sample with the addition of a 10-fold excess volume of ice-cold ethanol and the suspension kept at −20°C for one hour. Samples were centrifuged (16,000 × g) at 4°C for 10 minutes and the supernatants carefully removed by aspiration. Pellets were washed once with ice-cold ethanol and centrifuged. Each sample was resuspended in 50 µL of 50 mM ABC containing 1.0 mM calcium chloride (cell culture-grade, Sigma Aldrich, St. Louis, MO) and to each was added 1 µg trypsin (sequencing grade, Promega Corporation, Madison, WI) from a 1.0 µg/µL stock solution in the supplied storage solution. Digestion was allowed to proceed overnight at 37°C before samples were acidified with 200 µL of 5% formic acid (Optima grade, Thermo Fisher Scientific) containing 4% acetonitrile (ACN, LC-MS grade, Honeywell Burdick & Jackson, Morris Plains, NJ) in water (LC-MS grade, Honeywell Burdick & Jackson). Samples were freed from salts and impurities using C-18 cartridges prepared by adding 4 mgs of C-18-derivatized silica gel (25 µm particle size, 90 Å pore, SiliaSphere PC monomeric, SiliCycle, Quebec City, Canada) as a suspension in isopropanol (Optima grade, Thermo Fisher Scientific) to StageTips prepared in-house as previously described (20). Solvent was removed from all samples by vacuum centrifugation and resuspended in 5% formic acid containing 4% acetonitrile. Protein concentrations were equalized after measurement of UV spectra on a NanoDrop spectrophotometer (Thermo Fisher Scientific) and diluting samples to equal optical density at 280 nm.

### Instrument Methods

Nano-LC was performed on an Eksigent Ekspert nano-LC 400 system with nano-LC 425 pump (Eksigent Technologies, Dublin, CA) operating with Burdick & Jackson LC-MS-grade solvents. Channel A pumped 0.1% formic acid, while channel B ran 0.1% formic acid in acetonitrile. The separation column was equilibrated with 4% acetonitrile containing 0.1% formic acid at 300 nL/min. Flow rate was increased to 350 nL/min for sample loading. In all cases, approximately 1 µg of sample was loaded via the autosampler fitted with a 3.6 µL sample loop. After 10 minutes of loading at 350 nL/min flow rate and 4% acetonitrile, the loop was taken out of the flow path and the flow rate returned to 300 nL/min. A linear gradient to 30% B at 95 minutes was then executed, followed by a gradient to 50% B at 113 minutes, then a rapid gradient to 92% B at 115 minutes. Washing was performed to a run time of 120 minutes, at which point the system was returned to starting conditions and equilibration performed for 10 minutes before the next sample was run.

Chromatography was performed on a column packed in-house (50 µm × 180 mm) with C18-derivatized silica (ReproSil-Pur C18-AQ, 5 µm particle, 120 Å pore, Dr. Maisch Gmbh, Ammerbuch, Germany) terminating in a union with a silica emitter (PicoTip, New Objective, Littleton, MA) which interfaced the mass spectrometer (TripleTOF 6600+, Sciex, Framingham, MA) via a Nanospray III source operating at 2500 V and 150°C employing a nitrogen sheath gas at 4 psi and curtain gas running at 23 L/min.

LC-MS/MS data-dependent acquisition (DDA) experiments were performed in high-sensitivity mode by utilizing a MS parent ion scan from 400–1200 amu with an accumulation time of 250 msec. Criteria required for candidate selection in the MS/MS analyses were a minimum target peak intensity of 350 counts per second (cps), a charge state of 2–4, and selection of 60 candidates per cycle. After MS/MS analysis, also in high-sensitivity mode, candidate parent ions (50 mDa mass tolerance) were excluded from subsequent selection and sequencing for 20 seconds. MS/MS spectra were acquired from 100–1500 amu using an accumulation time of 75 msec and rolling collision energy determined by parameters defined by the manufacturer, Sciex. For DDA experiments, collision energy spread was not used. These experiments were used to generate spectral libraries for subsequent SWATH quantification analyses.

Data-independent acquisition (DIA) SWATH experiments were performed in high-sensitivity mode and started with a parent ion scan from 400–1200 amu, accumulation time of 250 msec. Windows of variable sizes were then determined using a calculator tool available online through Sciex (https://sciex.com/form-pages/sw-downloads-form?d=SWATH_Variable_Window_Calculator_v1.1.zip&asset=software&softwareProduct=SWATH%20Variable%20Window%20Calculator%20v1.1 The number of windows generated was kept below 60 so as to keep the duty cycle of analysis at or below 4 seconds. Accumulation time for all SWATH experiments was 50 msec and the mass range was 100–1250.

### Proteomics Data Analysis

Protein identification was performed using Protein Pilot software running the Paragon algorithm (Sciex). Data was searched against a human proteome database containing over 16,500 manually-annotated entries in FASTA format downloaded from the Uniprot website (https://www.uniprot.org). Searches were performed with cysteines modified (iodoacetamide). A target false-discovery rate (FDR) of 0.05 and a thorough ID search effort were selected for any analysis. A minimum of 95% confidence was used as a threshold for peptide identification as calculated by Protein Pilot. Relative quantification was performed using the SWATH processing microApp in PeakView software. Peak groups were extracted with a 99% peptide confidence threshold and 1% peptide FDR limit. SWATH chromatograms were extracted in 10-minute windows with fragment ion mass tolerance set to 75 ppm. Resulting protein quantitative peak areas were further analyzed using MarkerView software to compare relative quantities of all detected proteins between samples. Statistical analyses including t-tests and principal component analyses, were completed for data sets using Sciex MarkerView software. Significances of different protein relative abundances were determined via t-test (p < 0.05). Data was then exported as Microsoft Excel files. Human gene symbols for proteins with altered expression levels could then be entered into the STRING database for connectivity and enrichment analyses.

### Post-hoc Denaturing gel electrophoresis and immunoblotting

The total protein concentration of the CSF samples was determined with Protein Assay Dye Reagent (Bio-Rad, 5000006). The CSF samples were mixed with 6× SDS-PAGE loading buffer (0.35 M Tris-HCl, pH 6.8, 30% [v/v] glycerol, 12% [w/v] sodium dodecyl sulfate, 0.6 M dithiothreitol, and 0.06% [w/v] bromophenol blue) and heated at 94°C for 5 minutes. Equal total protein amounts were loaded on 4–12% Bis-Tris gradient protein gels (Thermo Fisher Scientific, NP0335BOX) and resolved by denaturing gel electrophoresis using MES SDS running buffer, pH 7.3 (Thermo Fisher Scientific, NP0002). The resolved proteins were transferred to nitrocellulose membranes with 0.2 µm pore size (Bio-Rad, 1704158). The membranes were stained with the Revert Total Protein Stain Kit (Li-Cor, 926–11016) and scanned with a Li-Cor Odyssey CLx Imaging System. The membranes were destained, followed by blocking with blocking buffer (5% non-fat dry milk, 50 mM Tris-HCl, 0.85% [w/v] NaCl, 0.05% [v/v] Tween-20, pH 7.5). The primary and secondary antibodies were applied in blocking buffer in 1:1,000 and 1:10,000 dilution, respectively. The primary antibodies were rabbit anti-CNDP1 (Atlas, HPA073283), and rabbit anti-RBP4 (HPA001641). The secondary antibody was IRDye 800CW goat anti-rabbit IgG (Li-Cor, 926–32211). The images were acquired on a Li-Cor Odyssey CLx Imaging System, and analyzed with the Li-Cor Image Studio software, version 5.2.5. The fluorescence intensities of the bands were normalized to total protein loading (as determined by Revert staining). An identical CSF sample was included on all gels for normalization.

## Results

**Figure 1:**
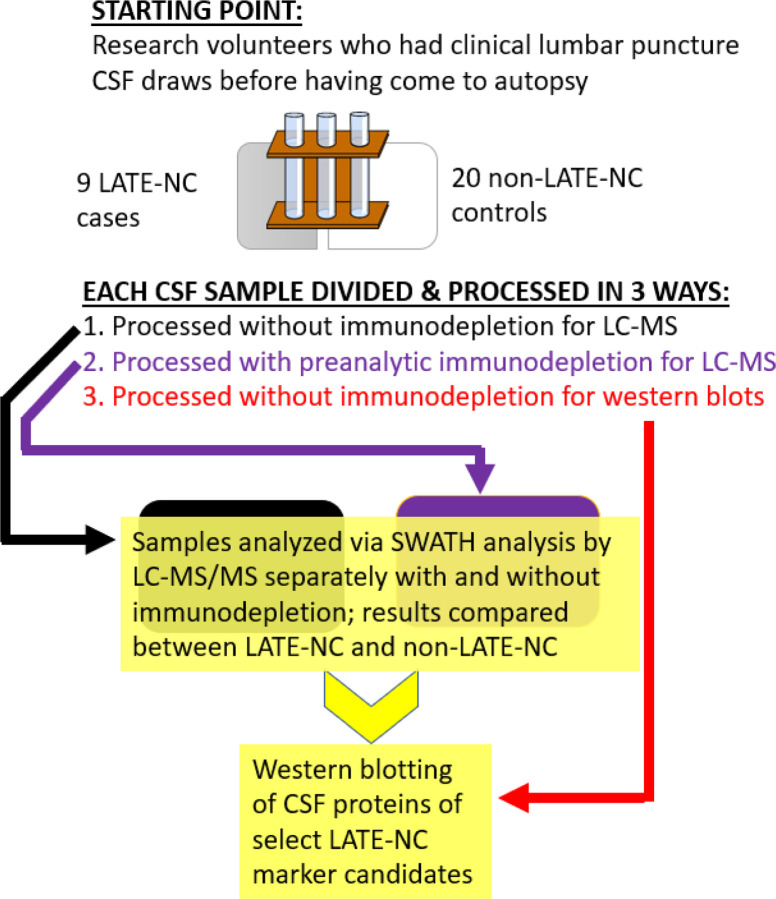
Work flow. A convenience sample of clinically obtained lumbar puncture cerebrospinal fluid (CSF) samples were analyzed from 29 older adults were the bases for this study. All the participants had come to autopsy and 9 had LATE-NC. CSF drawn during life was analyzed in separate preparations: From each CSF sample tube, 200 µl aliquots were depleted of highly abundant proteins with affinity spin columns. LC-MS/MS was performed as described above and protein identification was performed using Protein Pilot and PeakView SWATH microAPP and MarkerView software, also as described. Following data analyses, additional studies were performed using western blots on a subsample of proteins which showed differences between LATE-NC and non-LATE-NC cases.

Overall study design and workflow are represented schematically in Fig. 1. Clinical and pathological information about the included participants is shown in Table 1. The average age of the CSF draw was age 80.9 years whereas the average age of death was 85.1 years. Thus, the average interval between the CSF draw and death was slightly over 4 years. Of the 29 included subjects, 9 had autopsy-confirmed LATE-NC.

**Figure 2:**
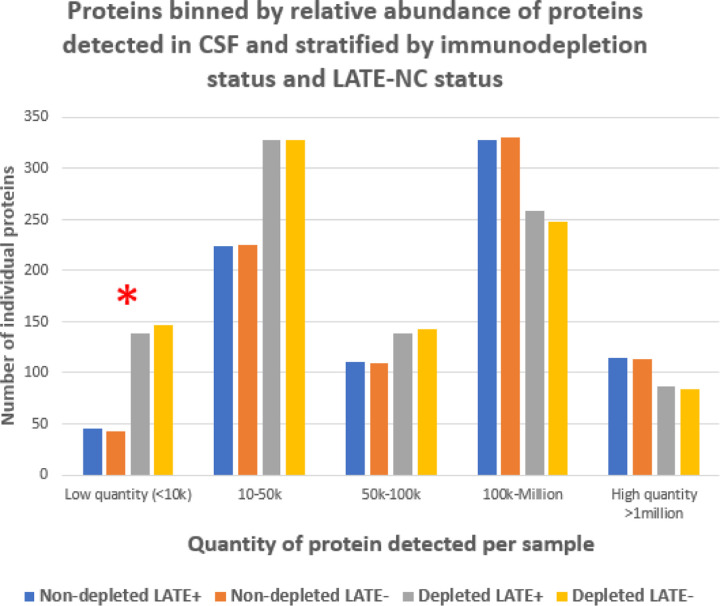
Results of two different pre-analytical preparations: immunodepleted and non-depleted samples. From each CSF sample tube, 200 µl aliquots were depleted of highly abundant proteins. The abundant proteins (albumin, IgA, IgD, IgE, IgG, kappa and lambda light chains, IgM, α1-acid glycoprotein, α1-antitrypsin, α2-macroglobulin, apolipoprotein A1, fibrinogen, haptoglobin, and transferrin) were depleted with the High-Select Top14 Abundant Protein Depletion Mini Spin Columns (Thermo Fisher Scientific, Cat. # A36370). Note that the result of the depletion step was to augment the number of different low-quantity proteins identified.

Two sets of samples were run separately – one set with the CSF depleted beforehand of abundant proteins (using affinity columns designed for this purpose as described in Methods); and, a set from the same cases but not depleted of abundant proteins. We hereafter refer to the samples depleted of abundant proteins as DEP and non-depleted samples as Non-DEP. As expected, the results in the DEP set indicated at least partially successful depletion of common CSF proteins (albumin, serotransferrin, etc; Fig. 2). The depletion resulted in capturing more than double the number of peptides corresponding to low-quantity proteins in the CSF (red asterisk in Fig. 2). Specifically, peptides corresponding to 820 different proteins were identified in the Non-DEP cases, and 949 different proteins in the DEP cases. In both DEP and Non-DEP samples, hereas some dementia-related proteins were detected (Aβ peptide and α-synuclein protein), others were not detected (TDP-43 and tau proteins). Raw results are shown in Supplemental Tables 1 and 2.

Analyses of mass spectrometry results (comparing between LATE-NC and non-LATE-NC cases) were performed separately for the DEP and Non-DEP samples. These results are presented to depict the top 20 detected proteins in order of lowest p-value (comparing LATE-NC and non-LATE-NC). Shown separately are results for proteins positively associated with LATE-NC in Non-DEP (Table 2); negatively associated with LATE-NC in Non-DEP (Table 3); positively associated with LATE-NC in the DEP (Table 4); and, negatively associated with LATE-NC in the DEP cases (Table 5). Results for all of the samples in both DEP and Non-DEP are presented in Supplemental Tables 1 and 2.

**Figure 3:**
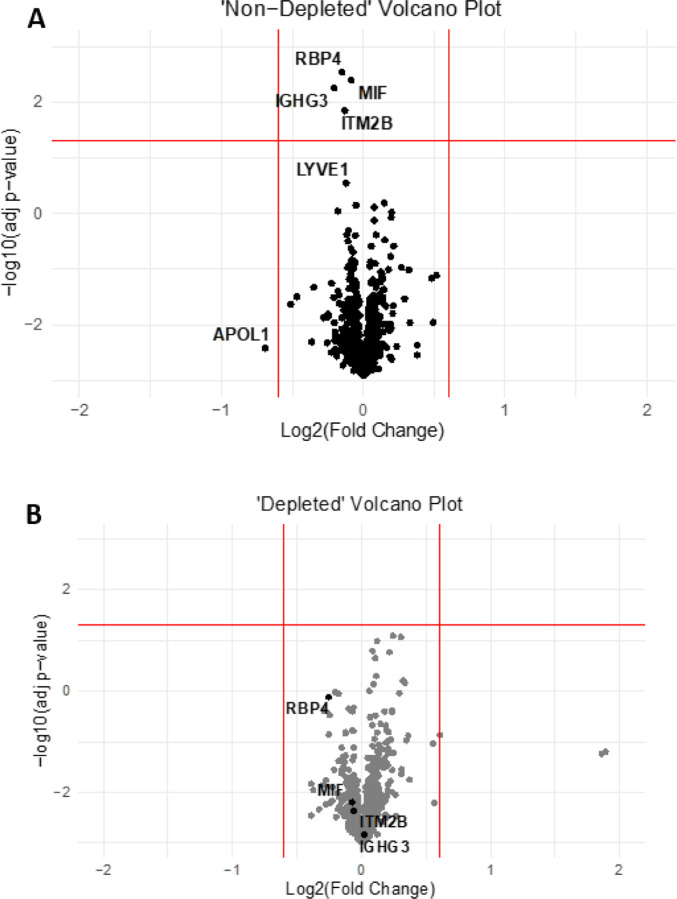
Volcano plots show the results for nondepleted (A) and affinity-depleted (B) samples. This chart enables display of both fold-change and p-values for all the analytes. Only a few proteins were found differentially in CSF from subjects with autopsy-confirmed LATE-NC. These included RBP4, MIF, IGHG3 and ITM2B, all of which were increased in non-depleted CSF from subjects with LATE-NC.

The only 4 protein analytes that satisfied the criteria for statistical significance at the p < 0.05 level following correction for multiple comparisons were in the Non-DEP samples: RBP4 (corrected p-value 0.003); MIF (0.004); IGHG3 (0.006); and, ITM2B (0.014). Each of these was detected at higher levels in the LATE-NC cases than in the non-LATE-NC cases (Fig. 3).

In the DEP samples, there were no statistically significant changes identified, after correcting for multiple comparisons (Tables 4, 5). However, the same protein (RBP4) that was found in the Non-DEP samples with the lowest p-value (comparing LATE-NC vs non-LATE-NC cases) also had the lowest-p-value for the DEP samples (p = 0.0014 before correcting for multiple comparisons).

Among the proteins with marginal trends for difference (LATE-NC vs non-LATE-NC cases) in the DEP samples was CNDP1 (p < 0.001 but did not survive correction for multiple comparisons). Detected CNDP1 was decreased in the LATE-NC samples. This protein had a similar marginal trend in the Non-DEP samples (nominal p = 0.002 in the same direction).

**Figure 4:**
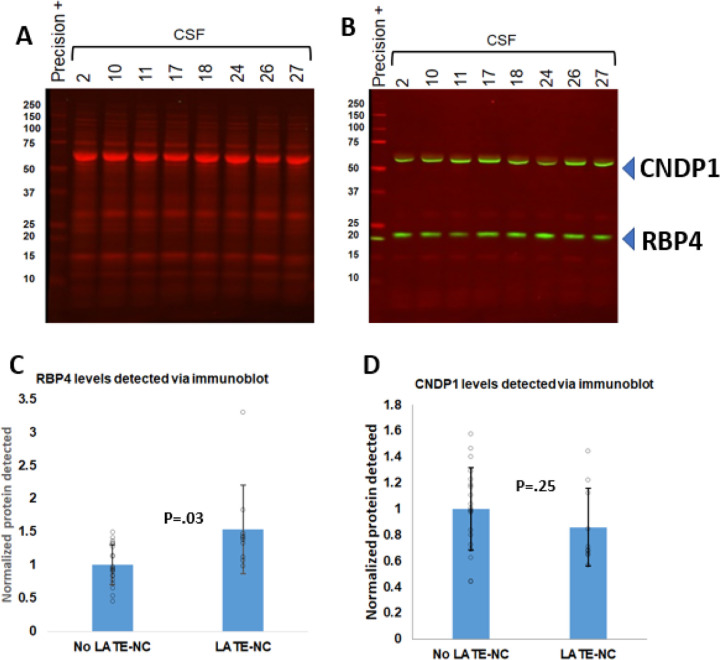
Post-hoc western blots of RBP4 and CNDP1 in non-depleted samples of CSF from the same 29 subjects whose CSF were used for LC-MS/MS analyses. Shown are two representative images (A, B) to depict the total protein staining (staining in red; A) as well as the specific bands blotted for RBP4 and CNDP1 (in green; B). As expected, the RBP4 antibody labeled a ~23kDa band, and the CNDP1 antibody labeled a ~57kDa band. The data across all 29 samples are charted in panels C (RBP4) and D (CNDP1). The results for both are in the same direction as shown with the LC-MS/MS studies but only the RBP4 results were statistically significant (p=0.03). Note, however, that there was substantial overlap between the analytical results for samples with and without LATE-NC.

Since there appeared to be relatively rubust signals for RBP4 (increased in LATE-NC), and CNDP1 (decreased in LATE-NC), in the CSF from subjects with LATE-NC in both the Non-DEP and DEP samples, we followed up the mass spectrometry findings with an assessment of immunoblots using the Non-Dep samples (Fig. 4). Here we used a commercially available antibody against RBP4. The RBP4 immunoblots showed the expected ~ 23 kDa band. The intensity of the bands were compared – LATE-NC vs non-LATE-NC using a digital image quantification tool. These results were compatible with the mass spectrometry, with LATE-NC samples having a higher average RBP4 signal (p = 0.03). There was not a statistically significant difference in CNDP1 (~ 57 kDa) levels comparing LATE-NC and non-LATE-NC samples. Moreover, even for RBP4, there was a large degree of overlap between the detected RBP4 levels in LATE-NC and non-LATE-NC cases. IGHG3 and ITM2B were not assessed via western blot because their mass spectrometry findings were relatively less robust.

## Discussion

The present exploratory study focused on a broad proteomic analysis of antemortem CSF that compared subjects with and without eventual autopsy-confirmed LATE-NC. For the first time we have identified two proteins (RBP4 and ITM2B) positively associated with LATE-NC and one protein (CNDP1) that is negatively associated with LATE-NC. The reported associations are intriguing and may open the door for a better understanding of the differences between LATE-NC and non-LATE-NC. Although the immunodepletion enabled detection of more low-expressed proteins (total 830 DEP versus 730 non-DEP), we did not find improved resolution of differentially detected peptides for the purposes of the current study.

RBP4 (retinol binding protein 4) is an adipokine member of the lipocalin protein family, and is the protein carrier for retinol (vitamin A) in blood (21, 22). RBP4 has previously been implicated as a biomarker for metabolic syndrome and cardiovascular disease (21–23). For predicting onset and progression of neurological disease, RBP4 has been suggested as a biomarker relevant to stroke and brain injury risk (24–30). A recent CSF study found that RBP4 increase was associated with blood-brain-barrier dysfunction (31). Collectively these results may further support the hypothesis that LATE-NC is linked with cerebral small blood vessel disease (12–15). By contrast, in a study of potential preclinical AD biomarkers, RBP4 in plasma was not associated with preclinical AD or incident dementia (32). We underscore that although RBP4 levels were increased in Non-DEP CSF (as validated by western blotting), and trended higher in DEP CSF samples in LATE-NC, there was overlap in the levels between LATE-NC indicating that it would not be an optimal candidate for a clinical biomarker. Still, rather than having direct implications in the clinical context, the RBP4/LATE-NC link may have disease-related mechanistic implications. Other clues to the involvement of the elevated RBP4 level in LATE-NC pathogenesis could be derived from the interaction partners of RBP4. The RBP4 protein was reported to interact with another CSF protein, transthyretin (33–35). Transthyretin was found to be neuroprotective in ischemia and AD (36). Proteomics screens identified the ferritin heavy chain as an RBP4 interaction partner (37, 38). The levels of ferritin in the CSF predict AD-related outcomes (39).

Another protein that trended to be increased in CSF of LATE-NC versus control cases was ITM2B (integral membrane protein 2B). This finding was only in Non-DEP samples, but we highlight the finding because of the significance of ITM2B as a dementia-related gene product. The *ITM2B* gene (also termed *BRI2*) encodes a transmembrane protein that can be cleaved near the C-terminus by furin-type proteases to produce a short peptide, via a mechanism analogous to the APP/Aβ processing pathway (40–42). Beyond resembling the APP/Aβ processing paradigm, ITM2B has also been shown to inhibit APP cleavage and Aβ fibril deposition(43–45). Furthermore, human genetic mutations which result in longer ITM2B protein C-terminal (and which increase the size and fibrillogenicity of the secreted peptide), are associated with two well-characterized neurogenerative diseases, familial British dementia and familial Danish dementia (40–42, 46–49). Both of these conditions show cerebral amyloid angiopathy (comprising ITM2B peptide fragment fibrils) (50), which is not a pathognomonic feature of LATE-NC.

As opposed to RBP4 and ITM2B proteins which tended to show increased CSF protein levels in LATE-NC, the amount of CNDP1 (Carnosine Dipeptidase 1) trended down for LATE-NC cases in DEP, Non-DEP, and western blot analyses. Although none of these results were statistically significant in these small samples of CSF, the finding of CNDP1 reduction is intriguing because a prior study found that CSF levels of CNDP1 were decreased in early AD (51). It is possible that the signal previously associated with AD (clinical) may in fact have been referent to the prevalent AD mimicking condition, LATE.

The novelty of this study was achieved from the use of a thoroughly clinically and pathologically characterized cohort. Other strengths of this study included excellent LC-MS resources and a well, and the parallel assessment with and without affinity-depletion of common proteins in the CSF. However, the present study did not identify a protein species in CSF that clearly differentiates between cases with and without LATE-NC.

There were some limitations to the current study. Most importantly, the sample sizes of the LATE-NC cases (particularly) and controls were small, as common in preliminary exploratory analyses. Additionally, the cases with LATE-NC were unintentionally biased toward also having ADNC (more likely to have higher Braak NFT stages). Further, all of the research participants studied were Caucasian limiting the generalizability of the study. Future studies in larger and more diverse study samples, enriched for LATE-NC without the confound of ADNC, are needed.

Another limitation in the present study is that there was not adequate sensitivity to detect some of the known low-level CSF proteins relevant to neurodegeneration, particularly TDP-43 or TMEM106B. TDP-43 protein (the critical pathognomonic feature of LATE-NC) has been identified in CSF by a number of other investigators. In the present study, the levels of Aβ trended slightly lower in the LATE-NC group, as expected given that ADNC was slightly higher in those participants. We emphasize that our a priori goals for this study were broader than evaluating protein levels of the known dementia-related proteins, and we also note that our methods did detect and quantify over 800 separate proteins in both DEP and Non-DEP. Among the interesting trends that noted in our analyses were findings referent to the proteins RBP4, CNDP1, and ITM2B.

In conclusion, we performed an exploratory assessment of CSF proteomes of autopsy-confirmed LATE-NC and non-LATE-NC cases from a community-based cohort. This study did not succeed to demonstrate a clear-cut proteomic fingerprint that distinguished the LATE-NC and non-LATE-NC cases. However, there were some intriguing findings that help expand our understanding of the potential molecular underpinnings of LATE-related brain changes. Further studies focused on biomarker development and discovery targeting RBP4, CNDP1, and ITM2B are needed.

## Figures and Tables

**Table 1.** Included subjects, with information about lumbar puncture timing, demographics, and pathology

Case #	LATE-NC status	Age at CSF draw (lumbar puncture)	Age at death	Sex	*APOE* haplo-type	Braak NFT Stage	CERAD Neuritic amyloid plaque	# gross infarcts
1	LATE-NC−	75	80	Female	e3/e3	V	C = Definite AD	1
2	LATE-NC−	71	75	Male	e3/e4	0	No	0
3	LATE-NC−	67	72	Male	e3/e4	VI	C = Definite AD	3
4	LATE-NC−	87	94	Female	e3/e3	II	A = CERAD possible	2
5	LATE-NC−	77	77	Male	e3/e4	II	No	5
6	LATE-NC−	86	87	Male	e3/e3	IV	No	0
7	LATE-NC−	79	84	Male	e3/e3	0	No	1
8	LATE-NC−	83	90	Female	e3/e3	II	No	0
9	LATE-NC−	84	89	Male	e3/e3	V	C = Definite AD	0
10	LATE-NC−	79	83	Female	e3/e4	I	No	0
11	LATE-NC−	83	84	Male	e2/e3	I	No	0
12	LATE-NC−	84	89	Male	e3/e4	III	B = CERAD probable	0
13	LATE-NC−	78	83	Female	e3/e3	V	C = Definite AD	1
14	LATE-NC−	74	81	Male	e4/e4	III	A = CERAD possible	4
15	LATE-NC−	72	72	Female	e3/e4	VI	C = Definite AD	0
16	LATE-NC−	71	73	Female	e3/e4	VI	C = Definite AD	0
17	LATE-NC−	85	87	Male	e4/e4	V	B = CERAD probable	0
18	LATE-NC−	78	86	Male	e3/e3	I	A = CERAD possible	1
19	LATE-NC−	93	99	Female	e3/e3	V	Yes	0
20	LATE-NC−	75	79	Male	e2/e3	I	No	1
21	LATE-NC+	87	90	Female	e3/e3	VI	C = Definite AD	1
22	LATE-NC+	75	81	Female	e3/e4	VI	C = Definite AD	3
23	LATE-NC+	86	92	Male	e3/e3	VI	C = Definite AD	1
24	LATE-NC+	87	89	Male	e3/e3	V	C = Definite AD	0
25	LATE-NC+	79	83	Female	e3/e3	1	No	0
26	LATE-NC+	89	89	Male	e3/e4	V	C = Definite AD	1
27	LATE-NC+	85	92	Female	e3/e3	V	B = CERAD probable	1
28	LATE-NC+	86	88	Male	e3/e3	V	C = Definite AD	3
29	LATE-NC+	93	100	Male	e3/e3	V	B = CERAD probable	0

**Table 2:** CSF proteins found at higher levels in LATE-NC than non-LATE-NC cases, in Non-DEP samples (top 15 scored by p-values)

Peptide Name	Peptide quantitation No LATE-NC	Peptide quantitation LATE-NC+	Fold Change	p-value	p-value (corrected)
sp|P02753|RET4 (RBP4)	3031484.01	4274051.94	1.41	3.53E-06	2.90E-03
sp|P14174|MIF	196259.95	238392.66	1.21	4.91E-06	4.03E-03
sp|P01860|IGHG3	547636.83	879147.65	1.61	6.84E-06	5.61E-03
sp|Q9Y287|ITM2B	71556.18	96429.79	1.35	1.73E-05	1.42E-02
sp|Q9Y5Y7|LYVE1	180260.89	238109.22	1.32	0.00035	NS
sp|P08571|CD14	1464104.02	1651576.47	1.13	0.00087	NS
sp|P02788|TRFL	71231.76	107851.01	1.51	0.00111	NS
sp|P36980|FHR2	94303.82	120185.35	1.27	0.00248	NS
sp|Q9Y279|VSIG4	43512.90	57083.99	1.31	0.00293	NS
sp|Q6EMK4|VASN	313080.97	355477.10	1.14	0.00303	NS
sp|Q9UBX7|KLK11	37981.93	48672.58	1.28	0.00385	NS
sp|Q12841|FSTL1	224963.54	275467.10	1.22	0.00516	NS
sp|Q96IY4|CBPB2	134648.50	158797.06	1.18	0.00613	NS
sp|P09382|LEG1	79095.80	95605.01	1.21	0.00836	NS
sp|Q9Y646|CBPQ	245078.60	281716.57	1.15	0.00892	NS

**Table 3:** CSF proteins found at lower levels in LATE-NC than non-LATE-NC cases, in Non-DEP samples (top 15 scored by p-values)

Peptide Name	Peptide quantitation No LATE-NC	Peptide quantitation LATE-NC+	Fold Change	p-value	p-value (corrected)
sp|P01857|IGHG1	65607862.55	46686136.73	0.71	0.00079	NS
sp|P30530|UFO	108745.50	91303.43	0.84	0.00095	NS
sp|P55285|CADH6	81734.85	51645.81	0.63	0.00116	NS
sp|A0A075B6K5|LV39	170471.30	108071.31	0.63	0.00143	NS
sp|Q96KN2|CNDP1	3130355.55	2619932.81	0.84	0.00164	NS
sp|O75882|ATRN	133569.37	108404.88	0.81	0.00297	NS
sp|P01619|KV320	4890768.79	3447030.14	0.70	0.00367	NS
sp|A0A0C4DH31|HV118	923304.39	562515.03	0.61	0.00469	NS
sp|P19021|AMD	206581.32	181115.71	0.88	0.00469	NS
sp|P15814|IGLL1	1269110.26	813914.78	0.64	0.00726	NS
sp|O15394|NCAM2	354031.59	314753.65	0.89	0.00893	NS
sp|Q7Z7M0|MEGF8	385118.93	317407.01	0.82	0.00973	NS
sp|Q8WVQ1|CANT1	110944.98	99687.32	0.90	0.01091	NS
sp|P01871|IGHM	936445.45	502282.43	0.54	0.01148	NS
sp|P26927|HGFL	29872.90	19828.73	0.66	0.01241	NS

**Table 4:** CSF proteins found at higher levels in LATE-NC than non-LATE-NC cases, in DEP samples (top 15 scored by p-values)

Peptide Name	Peptide quantitation No LATE-NC	Peptide quantitation LATE-NC+	Fold Change	p-value	p-value (corrected)
sp|Q15782|CH3L2	23556.70	37713.27	1.60	0.0011	NS
sp|P36980|FHR2	64732.78	97834.02	1.51	0.00119	NS
sp|P02753|RET4	2525576.61	4559489.06	1.81	0.00138	NS
(RBP4)					
sp|Q14118|DAG1	351505.51	407061.23	1.16	0.00225	NS
sp|P08572|CO4A2	17952.31	22614.98	1.26	0.00235	NS
sp|P02748|CO9	4823889.41	9427402.11	1.95	0.00262	NS
sp|P09871|C1S	2173045.90	2562222.11	1.18	0.00269	NS
sp|P14866|HNRPL	4118.80	7313.94	1.78	0.00314	NS
sp|P02747|C1QC	1057676.83	1244751.93	1.18	0.00625	NS
sp|P14618|KPYM	577898.47	668223.45	1.16	0.00641	NS
sp|Q03591|FHR1	208021.22	278022.89	1.34	0.00688	NS
sp|Q9NY97|B3GN2	7171.27	9586.11	1.34	0.00726	NS
sp|P00995|ISK1	8761.73	15685.66	1.79	0.00758	NS
sp|P07225|PROS	447766.49	516490.69	1.15	0.01381	NS
sp|P26927|HGFL	19295.41	27668.50	1.43	0.01384	NS

**Table 5:** CSF proteins found at lower levels in LATE-NC than non-LATE-NC cases, in DEP samples (top 15 scored by p-values)

Peptide Name	Peptide quantitation No LATE-NC	Peptide quantitation LATE-NC+	Fold Change	p-value	p-value (corrected)
sp|P01859|IGHG2	119766.70	68525.28	0.57	8.58E-05	NS
sp|P54802|ANAG	42713.31	21209.18	0.50	9.07E-05	NS
sp|Q96KN2|CNDP1	3055704.38	2328759.80	0.76	0.00011	NS
sp|P01210|PENK	342344.70	282305.39	0.82	0.00017	NS
sp|P01871|IGHM	14784.58	9007.90	0.61	0.00018	NS
sp|P27824|CALX	34015.41	26600.03	0.78	0.00024	NS
sp|P01303|NPY	82010.78	63487.10	0.77	0.00053	NS
sp|P61978|HNRPK	13930.75	6671.30	0.48	0.00066	NS
sp|P05387|RLA2	48237.35	22337.84	0.46	0.00073	NS
sp|O95502|NPTXR	645838.05	523772.64	0.81	0.00078	NS
sp|Q13740|CD166	455061.34	396976.34	0.87	0.00106	NS
sp|P43307|SSRA	10182.99	5191.28	0.51	0.00117	NS
sp|P22626|ROA2	52703.24	30607.02	0.58	0.00259	NS
sp|P60709|ACTB	192881.04	128069.11	0.66	0.00268	NS
sp|P04843|RPN1	9928.83	5753.58	0.58	0.00273	NS

## Abbreviations

ABC: phosphate buffered saline
ADNC: Alzheimer’s disease neuropathologic changes
BCA: Bicinchoninic acid
CAA: Cerebral amyloid angiopathy
CSF: cerebrospinal fluid
DDA: data-dependent acquisition
DEP: CSF samples depleted using chromatographic minicoloumns
FTD: Frontotemporal dementia
LATE-NC: Limbic predominant age-related neuropathologic changes
LBP: Lewy body pathology
NFT: neurofibrillary tangle
LC-MS/MS (Nano-LC): Liquid Chromatography Tandem Mass Spectrometry
PBS: phosphate buffered saline
SWATH: Sequential Window Acquisition of All Theoretical Mass Spectra
TCEP: Tris (2-carboxyethyl) phosphine
UK-ADRC: University of Kentucky Alzheimer’s Disease Research Center
